# Ocular Involvement as a Key Marker of Systemic Disease in Dogs Naturally Infected with *Leishmania infantum*: Clinical, Laboratory, and Histopathological Insights

**DOI:** 10.3390/pathogens15020217

**Published:** 2026-02-14

**Authors:** Caroline Magalhães-Cunha, Ana Lúcia Abreu-Silva, Marcelo Pelajo-Machado, Celeste da Silva Freitas de Souza, Karen Lebreiro dos Santos, Lucas Almeida Zangirolami, Flávia de Oliveira Cardoso, Kátia da Silva Calabrese

**Affiliations:** 1 Laboratório de Protozoologia, Instituto Oswaldo Cruz, Fiocruz, Av. Brasil, 4.365, Manguinhos, Rio de Janeiro 21040-360, RJ, Brazil; carolmcvet@gmail.com (C.M.-C.); csfsouza@ioc.fiocruz.br (C.d.S.F.d.S.); karenlebreiro03@gmail.com (K.L.d.S.); lucas.zangirolami1@gmail.com (L.A.Z.); calabrese@ioc.fiocruz.br (K.d.S.C.); 2 Programa de Pós-graduação em Medicina Tropical, Instituto Oswaldo Cruz, Fiocruz, Av. Brasil, 4.365, Manguinhos, Rio de Janeiro 21040-360, RJ, Brazil; 3 Laboratório de Patologia Veterinária, Departamento de Patologia, Universidade Estadual do Maranhão, Av. Lourenço Vieira da Silva, 1000, Jardim São Cristóvão, São Luís 65055-310, MA, Brazil; abreusilva.ana@gmail.com; 4 Laboratório de Medicina Experimental e Saúde, Instituto Oswaldo Cruz, Fiocruz, Av. Brasil, 4.365, Manguinhos, Rio de Janeiro 21040-360, RJ, Brazil; mpelajo@ioc.fiocruz.br; 5 Programa de Pós-graduação em Biologia Parasitária, Instituto Oswaldo Cruz, Fiocruz, Av. Brasil, 4.365, Manguinhos, Rio de Janeiro 21040-360, RJ, Brazil

**Keywords:** *Leishmania infantum*, dog, canine visceral leishmaniasis, ocular leishmaniasis

## Abstract

Canine visceral leishmaniasis (CVL), caused by *Leishmania infantum*, is a multisystemic disease in which ocular involvement is frequent but often underestimated. This study aimed to comprehensively evaluate the clinical, ophthalmological, parasitological, hematological, biochemical, and histopathological alterations in dogs naturally infected with *L. infantum* from an endemic area of northeastern Brazil, with special emphasis on the relationship between ocular manifestations and systemic disease. Twenty-five symptomatic dogs were evaluated through clinical and ophthalmological examinations, parasitological culture, PCR, laboratory analyses, and histopathology of ocular and periocular tissues. Ocular alterations were observed in 80% of the animals, predominantly bilateral and frequently associated with multiple concurrent lesions, including ocular discharge, conjunctivitis, blepharitis, uveitis, and corneal opacity. Functional ophthalmological tests revealed keratoconjunctivitis sicca and corneal ulcers in a substantial proportion of dogs. Hematological abnormalities were highly prevalent, particularly anemia and thrombocytopenia. Comparative analysis demonstrated that dogs with ocular involvement exhibited significantly higher leukocyte counts and segmented neutrophils, as well as increased AST levels, indicating an enhanced systemic inflammatory response. Histopathological examination revealed intense plasmacytic inflammatory infiltrates and the presence of amastigote forms in ocular and periocular tissues, indicating that both immune-mediated and parasite-driven mechanisms could be involved in disease pathogenesis. Collectively, these findings underscore ocular involvement as a clinically relevant manifestation of CVL and reinforce the importance of routine ophthalmological evaluation in clinical management.

## 1. Introduction

Canine visceral leishmaniasis (CVL) is a chronic and severe systemic disease caused by protozoan parasites belonging to the subfamily Leishmaniinae [1]. In Brazil, CVL is caused by *Leishmania infantum* [2] and transmitted by sandflies of the genus *Lutzomyia*, primarily, *Lutzomyia longipalpis* [3,4]. Dogs play a central role in the transmission cycle of human visceral leishmaniasis (HVL), acting as the main domestic reservoirs in urban environments [3,5,6].

The clinical presentation of CVL is often nonspecific and highly variable, frequently overlapping with other infectious or inflammatory conditions. Disease expression is influenced by multiple factors, including parasite burden, host–parasite interactions, duration of exposure to the vector, co-infections, immune status, and host genetic background [7,8,9,10]. After infection, parasites are phagocytosed and differentiate within macrophages. These infected cells subsequently migrate to regional lymph nodes and disseminate to other organs [11]. As a result, CVL is characterized by multisystemic involvement, affecting several organs, including ocular tissues and associated structures [12,13,14,15].

Dogs naturally infected with *L. infantum* commonly exhibit pathological changes in multiple organs, reflecting the systemic nature of the infection and its typically prolonged incubation period. The most frequently reported clinical alterations include skin lesions, cachexia, lymphadenopathy, hepatosplenomegaly, and renal involvement [16,17,18,19,20]. Within this multisystemic context, ocular alterations have been described as part of the clinical spectrum observed in dogs with CVL.

Ocular manifestations have been reported in dogs with CVL and are commonly observed in association with systemic alterations, although they may also occur in the absence of other overt clinical signs [7,14,21]. The most frequently described ocular changes include blepharitis, keratoconjunctivitis, and uveitis, with reported prevalence ranging from 16% to 80% among infected dogs [7,22,23]. The severity and anatomical distribution of these alterations vary, ranging from mild periocular changes to severe involvement of intraocular structures, which may be accompanied by visual impairment. In some cases, ocular signs constitute the main clinical complaint reported by owners and may be identified before other systemic manifestations are clinically evident [22,24,25,26].

Despite the reported frequency of ocular involvement in CVL, relatively few studies have systematically characterized ocular alterations in naturally infected dogs. Most available data derive from isolated case reports or studies involving small cohorts, which limits a comprehensive understanding of the spectrum of ocular pathology associated with CVL. Consequently, further studies are needed to better define the nature and extent of ocular lesions in this disease. Such investigations contribute to a more detailed characterization of clinical findings in dogs with CVL, ultimately improving clinical management and therapeutic decision-making.

## 2. Materials and Methods

### 2.1. Animals

All animals included in this study were seropositive for *Leishmania infantum* and were collected by the Municipal Zoonosis Control Department in the Cidade Operária neighborhood (Tirirical district), São Luís, Maranhão, Brazil, following a seroepidemiological survey. In accordance with the guidelines established by the Brazilian Ministry of Health for the management of canine visceral leishmaniasis (CVL) [27] these animals were designated for compulsory euthanasia. A total of 25 mongrel dogs (14 females and 11 males), aged over six months, were included. All animals were naturally infected with *L. infantum* and presented positive results in at least two serological tests for CVL: a rapid immunochromatographic test (DPP^®^ CVL, Bio-Manguinhos, Rio de Janeiro, Brazil) and an enzyme-linked immunosorbent assay (ELISA-EIE™, Bio-Manguinhos, Rio de Janeiro, Brazil) and/or the infection was further confirmed by parasitological analysis or by polymerase chain reaction (PCR).

### 2.2. Clinical and Ophthalmic Examination

A complete clinical evaluation was performed on all animals. Clinical signs compatible with CVL included skin lesions, alopecia, cachexia, hepatosplenomegaly, lymphadenomegaly, and onychogryphosis or onychodystrophy. Ocular abnormalities assessed included mucoid, purulent, or hemorrhagic ocular discharge, conjunctivitis, blepharitis, uveitis, and corneal opacity.

All clinical and ophthalmic examinations were performed by a single professional, a veterinarian specialized in ophthalmology, using a standardized protocol consistently applied to all animals included in the study.

### 2.3. Ophthalmological Tests

Schirmer’s tear test (STT) (Ophthalmos, São Paulo, Brazil) was performed to assess tear production and assist in the diagnosis of keratoconjunctivitis sicca (KCS), following the manufacturer’s instructions. Tear production values > 10 mm/min were considered normal; values between 5 and 10 mm/min were deemed suspicious, and values below ˂5 mm/min were diagnostic for KCS [28].

Fluorescein staining was employed to detect corneal epithelial defects. One drop of ophthalmic fluorescein dye (Ophthalmos, São Paulo, Brazil) was instilled in each eye, and excess dye was rinsed off. Areas of epithelial disruption were visualized as bright green under cobalt blue light.

### 2.4. Sample Collection and Laboratory Procedures

Blood samples (5 mL) were collected from the cephalic vein using 21-gauge needles. Samples containing EDTA were used for hematologic analysis, whereas those without anticoagulant were used for biochemical assays. Serum levels of alanine aminotransferase (ALT), aspartate aminotransferase (AST), total protein, albumin, globulin, urea, and creatinine were determined. All analyses were conducted at the Flávia Uchôa Veterinary Clinical Laboratory.

Bone marrow samples were obtained under anesthesia, induced with intramuscular administration of xylazine (0.1–0.15 mL/kg) combined with ketamine (0.2–0.3 mL/kg). Bone marrow smears were stained with Giemsa and examined under light microscopy at 100× magnification.

Following whole blood and blood marrow collection, animals were euthanized via intravenous administration of thiopental disodium (2.5%; 3–15 mg/kg) followed by potassium chloride (100 mg/kg), as recommended [29]. Tissue samples were collected from eyelids, globes, spleen, as well as aspirates of aqueous humor and spleen. Aspirates were inoculated into NNN medium [30] and Schneider’s medium supplemented with 10% fetal bovine serum. Cultures were incubated at 26 °C and examined weekly. Positive cultures were submitted to the *Leishmania* Collection (CLIOC), Oswaldo Cruz Institute (IOC/Fiocruz), Rio de Janeiro, Brazil, for species identification via multilocus enzyme electrophoresis (MLEE).

### 2.5. Polymerase Chain Reaction (PCR) Assay

PCR was performed on samples from which parasites could not be visualized or isolated. Total DNA was extracted from spleen fragments (3–5 mm), that had been previously stored at −20 °C in RNAlater^®^ Solution (Applied Biosystems, Foster City, CA, USA). Extraction involved suspending the tissue in 500 µL of lysis buffer (50 mM Tris-HCl, 10 mM NaCl, 5 mM EDTA, 0.5% SDS) containing proteinase K (20 mg/mL), followed by overnight incubation at 56 °C. DNA was then purified using the standard phenol/chloroform method [31]. DNA purity and quality (assessed by 260/280 and 260/230 absorbance ratios, with all samples meeting standard criteria of 260/280: 1.8–2.0 and 260/230: >2.0) and concentration were determined using a NanoDrop 2000c spectrophotometer (Thermo Fisher Scientific, Wilmington, DE, USA). DNA integrity was confirmed by successful amplification of the canine GAPDH gene in PCR assays.

PCR amplification targeted *Leishmania* kinetoplast DNA (minicircle) using the primers: forward 5′-CCT ATT TTA CAC CAA CCC CCA GT—3′and reverse 5′-GGG TAG GGG CGT TCT GCG AAA-3′; and the canine GAPDH gene using forward: 5′-TCA ACG GAT TTG GCC GTA TTG G-3′ and reverse: 5′-TGA AGG GGT CAT TGA TGG CG-3′) as described previously [32,33]. PCR was performed in 25 µL reaction volumes containing 1× reaction buffer (100 mM Tris-HCl, pH 8.3; 500 mM KCl), 4 mM MgCl_2_, 0.2 mM dNTPs, 250 nM (GAPDH) or 400 nM (*Leishmania*) primers, 1U/µL Taq polymerase, 100 ng of genomic DNA, and nuclease-free water. All PCRs were performed in duplicate to ensure reliability and reproducibility.

Amplification conditions were:

GAPDH: 95 °C for 10 min; 40 cycles of 95 °C for 30 s, 60 °C for 1 min and 72 °C for 30 s; final extension at 72 °C for 5 min.

*Leishmania* minicircle: 95 °C for 5 min; 35 cycles of 95 °C for 30 s, 54 °C for 45 s, and 72 °C for 30 s; final extension at 72 °C for 10 min.

PCR was conducted using a GeneAmp^®^ 9600 Thermal Cycler (Applied Biosystems, Foster City, CA, USA). Amplified products (5 µL) were subjected to 2% agarose gel electrophoresis, stained with GelRedTM (Biotium, CA, USA), and visualized under UV transillumination.

### 2.6. Histopathology

Fragments of the globe and ocular adnexa were fixed in 10% neutral-buffered formalin, embedded in paraffin, and sectioned at 5 μm. Sections were stained with Hematoxylin and Eosin (H&E) for histopathological examination. Histopathological evaluations were conducted blind to clinical findings by a single observer.

### 2.7. Statistical Analysis

The data were expressed by mean ± standard deviation (SD) and analyzed statistically by Mann–Whitney and Student’s *t*-tests (*p* = 0.05). The choice between Student’s *t*-test and the Mann–Whitney test was based on data distribution assessed using the Shapiro–Wilk normality test, where *p* > 0.05 was considered indicative of normal distribution. The analyses were performed with the software GraphPad Prism 9.

## 3. Results

### 3.1. Clinical Examination

**Figure 1:**
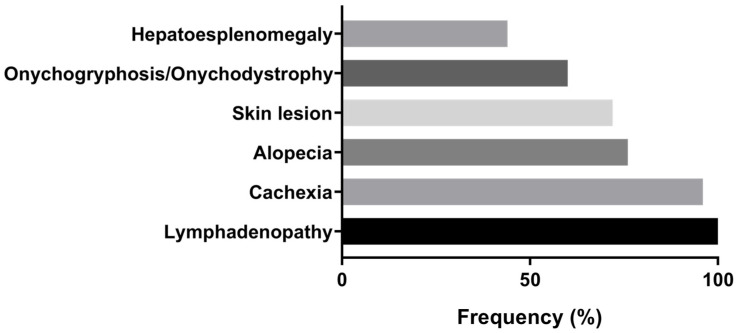
Frequency of systemic clinical manifestations observed during physical examination of dogs naturally infected with *Leishmania infantum* (*n* = 25) in São Luís, Brazil.

Based on the evaluation of clinical signs, all examined animals were classified as symptomatic when they exhibited two or more clinical characteristic manifestations of CVL (Supplementary Table S1). Lymphadenopathy (*n* = 25; 100%) was the most frequent clinical finding, followed by cachexy (*n* = 24; 96%), alopecia (*n* = 19; 76%), skin lesions (*n* = 18; 72%), onychogryphosis/onychodystrophy (*n* = 15; 60%) and hepatosplenomegaly (*n* = 11; 44%), respectively (Figure 1).

**Figure 2:**
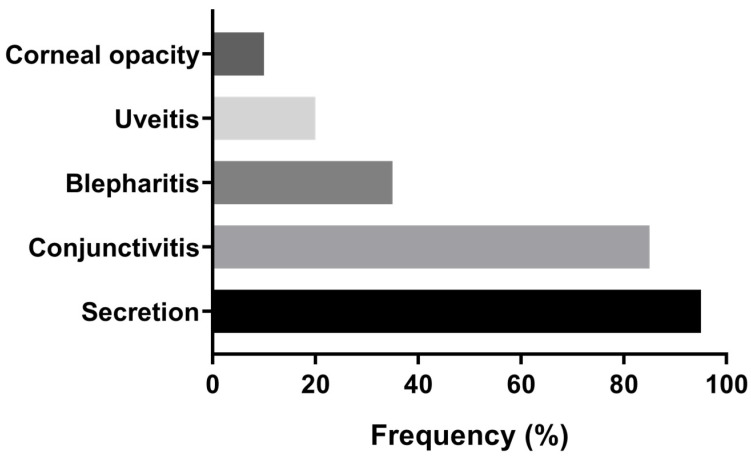
Frequency of ophthalmological alterations identified in dogs with ocular involvement (*n* = 20) naturally infected with *Leishmania infantum* in São Luís, Brazil.

Ophthalmological assessment revealed that 80% (20/25) of the dogs presented bilateral ocular alterations. Among these, 90% (18/20) exhibited more than one type of lesion, and 50% (10/20) showed at least two distinct alterations. Notably, an association between ocular discharge and conjunctivitis was observed in 80% (16/20) of the affected animals (Supplementary Table S1). Ocular discharge (mucopurulent, purulent, or hemorrhagic) was the most common finding, present in 95% (19/20) of cases, followed by conjunctivitis (17/20; 85%), blepharitis (7/20; 35%), uveitis (4/20; 20%) and corneal opacity (2/20; 10%) (Figure 2).

### 3.2. Isolation and Characterization of Parasites

Parasite isolation in culture was successful in 14 animals (14/25; 56%). Among these, seven isolates (7/14; 50%) were obtained from bone marrow, six (6/14; 43%) from spleen samples, and one (1/14; 7%) from aqueous humor. It was not possible to isolate parasites in tissues of 11 animals (11/25; 44%), due to bacterial and fungal contamination, although it was possible to observe promastigote forms in three cultures (3/25; 12%), and in three cases (3/25; 18%), amastigotes were detected in bone marrow smears (Supplementary Table S4).

The parasites isolated from the samples were characterized as *Leishmania infantum*, according to the species identification report provided by CLIOC. One sample was excluded from the analysis due to excessive bacterial contamination.

### 3.3. PCR

PCR was performed on six samples from animals that tested negative in parasitological assays (bone marrow smear and parasite isolation in culture).

**Figure 3:**
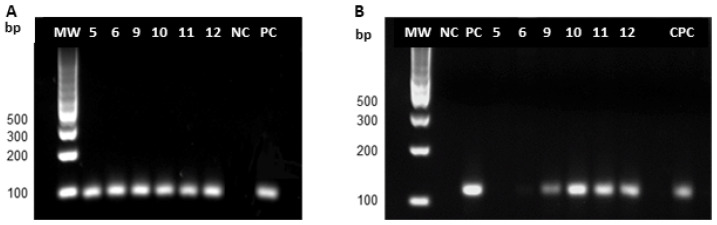
Agarose gel electrophoresis of PCR products. (**A**) GAPDH; (**B**) Minicircle. MW, molecular weight marker; bp, base pairs; NC, negative control; PC, positive control (*Leishmania infantum*); CPC, positive control from an infected dog; lanes 5, 6, 9, 10, 11, 12, dogs with both negative parasitological isolation and bone marrow smears.

Leishmania DNA, corresponding to the ~120 bp amplification product of the minicircle PCR, was detected in four animals. Two samples tested negative (Figure 3B).

### 3.4. Ophthalmological Tests

Schirmer’s tear test revealed reduced tear production in 32% (8/25) of the animals, with values ranging from 1.5 to 6.0 mm/min, consistent with KCS.

The fluorescein test was positive in 28% (7/25) of the animals, indicating the presence of corneal ulcers. When the results of both tests were combined, four animals (16%) tested positive in both assessments.

### 3.5. Hematological and Biochemical Tests

Hematological abnormalities were detected in almost all animals evaluated (96%; *n* = 24) (Supplementary Table S2). Among red blood cell parameters, anemia was the most frequent alteration, observed in 92% (23/25) of the animals and characterized by decreased erythrocyte counts, hemoglobin levels, and/or hematocrit values. Thrombocytopenia was also common, affecting 52% (13/25) of the animals (Table 1).

Regarding changes in the leukogram, reduced leukocyte counts were observed in 11 of 25 dogs (44%). Reductions in specific leukocyte subsets were also common, including monocytopenia (13/25; 52%), lymphopenia (10/25; 40%), eosinopenia (10/25; 40%), and neutropenia (7/25; 28%). Other abnormalities as neutrophilia (20%; 5/25), with a left shift in two cases (8%), leukocytosis (16%; 4/25), monocytosis (8%; 2/25), eosinophilia (4%; 1/25), and lymphocytosis (4%; 1/25) (Table 1), were also observed.

Biochemical profiles demonstrated heterogeneous patterns across the evaluated parameters (Supplementary Table S3). Regarding biochemical markers, AST and ALT were generally within normal limits for most animals (72–88%), with only a minority showing deviations. Creatinine levels were normal for 52% of individuals, although 12% exceeded reference values. Urea concentrations were within expected limits in 84% of the population, with 16% above normal. Total protein levels showed greater dispersion, with only 36% within the reference interval and 40% above it, largely due to elevated globulin concentrations: 60% of animals had normal globulin values, while 40% were above reference limits. In contrast, albumin levels were below reference values in 60% of the cohort, indicating frequent hypoalbuminemia. (Table 1).

Table 2 presents a comparison of hematological and biochemical parameters between dogs with ocular involvement (*n* = 20) and those without ocular involvement (*n* = 5), based on the presence of ocular abnormalities described in Supplementary Table S1. When group means were compared, dogs with ocular involvement exhibited significantly higher total leukocyte counts than dogs without ocular involvement (WBC: 10,383 ± 7570 vs. 4320 ± 3065 cells/µL; *p* = 0.0489), mainly driven by a marked increase in segmented neutrophils (7986 ± 6095 vs. 2817 ± 1509 cells/µL; *p* = 0.0192). No significant differences were observed between groups for lymphocyte, monocyte, eosinophil, or band neutrophil counts (Table 2).

Erythrogram parameters, including red blood cell count, hemoglobin concentration, hematocrit, and platelet count, did not differ significantly between dogs with and without ocular involvement (*p* > 0.05), indicating the absence of marked anemia or thrombocytopenia associated with ocular manifestations in this cohort.

Regarding biochemical parameters, dogs with ocular involvement presented significantly higher AST levels compared to dogs without ocular involvement (47.08 ± 20.23 vs. 26.84 ± 15.05 U/L; *p* = 0.0485). Although ALT levels tended to be higher in dogs without ocular involvement, this difference did not reach statistical significance (*p* = 0.0723). Renal function markers (creatinine and urea) and protein profile (total proteins, albumin, and globulin) showed no statistically significant differences between groups.

### 3.6. Histopathology

**Figure 4:**
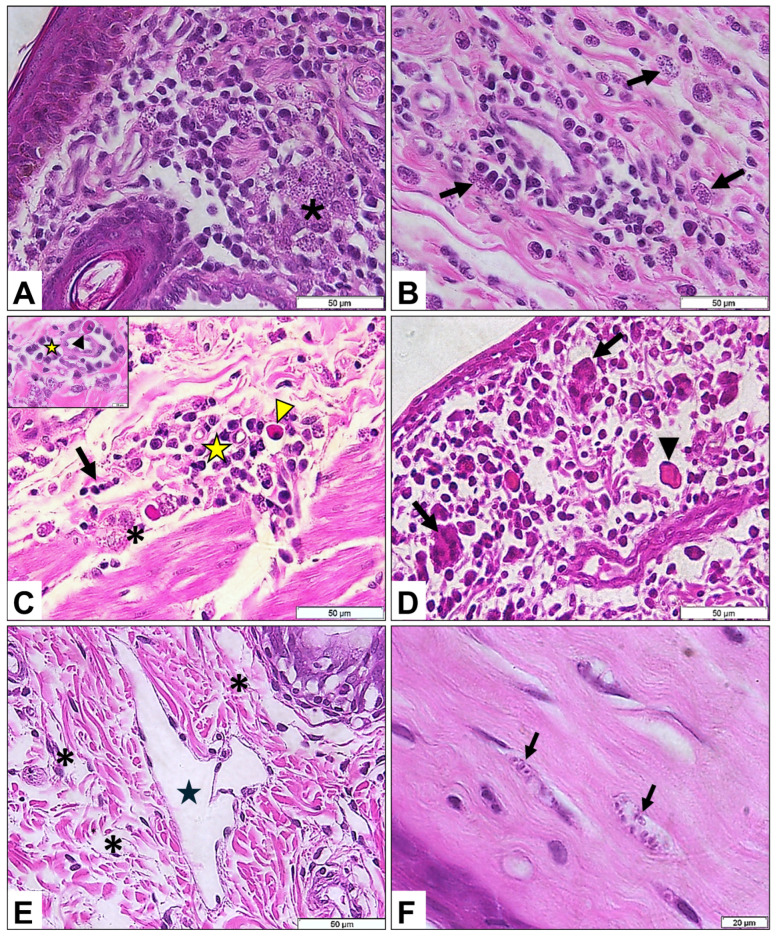
—Histopathological alterations of Dogs Naturally Infected with *Leishmania infantum* in São Luís, Brazil. (**A**)—diffuse plasmacytic inflammatory infiltrate associated with various amastigote forms in the interstitium (asterisk); (**B**)—amastigotes distributed in the interstitium (arrows); (**C**)—neutrophils (arrow) and Mott cells (arrowhead) associated with plasmacytic inflammatory infiltration (star) with the presence of amastigote forms (asterisk); (**D**)—giant cells (arrow) and Mott cells (arrowhead) associated with plasmacytic inflammatory infiltration (arrow); (**E**)—lymphatic vessel ectasia with valve (star) and edema (asterisk); (**F**)—amastigote forms within fibers (arrows).

Histopathological changes were identified in 76% (19/25) of the eyelid samples analyzed. The most frequent alteration was a plasmacytic inflammatory infiltrate involving the full thickness of the dermis, with intensity ranging from mild to severe (Figure 4A). Amastigote forms were detected in 21% (4/19) of the affected eyelids, either free within the stroma or contained within macrophages (Figure 4A,B). In addition, neutrophils and Mott cells were associated with infiltrates in 16% (3/25) of the animals (Figure 4C), and multinucleated giant cells were observed in one case (5%; 1/25) (Figure 4D). In the same animal, lymphatic vessel ectasia with valvular structures and eyelid edema were also noted (Figure 4E).

Beyond the eyelids, plasmacytic inflammatory infiltrates were also observed in the ocular and periocular tissues, including the cornea (*n* = 4), choroid (*n* = 3), sclera (*n* = 11), and periorbital muscles (*n* = 11). Amastigote forms were identified in the corneal stroma and retro-orbital musculature of two animals (Figure 4F).

The overview of all diagnostic results, including the number of animals testing positive by each applied diagnostic method (serological assays, parasitological examination, PCR, ophthalmological assessment, and histopathology), is presented in Supplementary Table S4.

## 4. Discussion

This study provides a comprehensive assessment of clinical, ophthalmological, hematological, biochemical, parasitological, and histopathological alterations in dogs naturally infected with *Leishmania infantum* in an endemic region of Brazil. The integration of parasitological confirmation with systemic and ocular findings highlights the multisystemic nature of CVL, as observed in this cohort. This observation is consistent with previous clinical and immunopathological descriptions reporting heterogeneous involvement of multiple organ systems in canine infection [34]. Inflammatory markers and immune alterations were frequently identified in symptomatic animals, supporting their recognized association with active disease, although no longitudinal or prognostic inferences can be drawn from the present data.

All dogs included in this study were symptomatic, with lymphadenopathy, cachexia, alopecia, and skin lesions representing the most frequent clinical findings. These manifestations are consistent with commonly reported clinical presentations of CVL and reflect chronic inflammatory and immune-complex-mediated processes described in the literature [35,36]. The frequent occurrence of onychogryphosis and hepatosplenomegaly further illustrates the systemic involvement observed in these animals, in agreement with previous studies [37]. Associations between clinical findings and hematological or biochemical alterations were observed, consistent with earlier reports linking anemia, hyperglobulinemia, and protein profile changes to clinical expression of CVL [38]. Anemia (92%) and thrombocytopenia (52%) were prevalent in this cohort and may reflect mechanisms such as chronic inflammation, splenic sequestration, and bone marrow involvement, as described in canine leishmaniasis [39].

Ocular alterations were identified in 80% of the evaluated dogs and included conjunctivitis, ocular discharge, blepharitis, uveitis, and corneal opacity. These findings are consistent with previous descriptions of ocular involvement in CVL [40,41] and established veterinary ophthalmology references [13,22]. Multiple ocular lesions frequently co-occurred, indicating a persistent inflammatory process affecting different ocular structures. Keratoconjunctivitis sicca was identified in approximately one-third of the animals, while corneal ulcers were observed in nearly 30%. These alterations may be associated with lacrimal gland dysfunction, inflammation-related tear film instability, or periocular tissue involvement. The detection of amastigotes in periocular musculature supports parasite presence in ocular adnexa and provides pathological context for the inflammatory changes observed. Similar findings have been reported in naturally infected dogs, in which parasite-induced myositis leads to periorbital muscle atrophy and subsequent ocular exposure [7].

Ocular manifestations have been reported in dogs even in the absence of overt systemic clinical signs [22]. Localized inflammatory responses triggered by *Leishmania* spp. within ocular tissues may lead to alterations such as uveitis, chronic conjunctivitis, keratoconjunctivitis, corneal opacities, and blepharitis. These alterations have frequently been described as isolated findings, particularly during the early phases of infection [22,40], highlighting ophthalmic examination as a critical component for early diagnostic suspicion. Notably, ocular abnormalities may precede the development of classical systemic clinical signs; therefore, the absence of generalized manifestations does not rule out leishmaniasis, especially in dogs residing in endemic areas [19]. In this context, the persistence of ocular signs should prompt the inclusion of leishmaniasis in the differential diagnosis and the implementation of specific complementary diagnostic tests, thereby facilitating early detection and appropriate clinical management [19,42]. However, although ocular alterations may occur prior to other clinical manifestations, the present study does not evaluate temporal relationships or diagnostic performance. Consequently, while ocular findings represent an important component of the clinical spectrum of CVL, their presence alone should not be considered as predictive or diagnostic in the absence of confirmatory testing [19,42].

Histopathological analysis revealed intense plasmacytic infiltrates in the eyelids, sclera, periocular musculature, and cornea, frequently accompanied by Mott cells, neutrophils, and occasional multinucleated giant cells. The identification of amastigotes in ocular and periocular tissues supports the coexistence of direct parasitism and immune-mediated mechanisms in lesion development, consistent with previous descriptions of ocular leishmaniasis [7,43]. Parasite persistence in these tissues may contribute to the chronic inflammatory changes observed; however, conclusions regarding disease persistence or recurrence cannot be established within the scope of this study. Parasite isolation was successful in over half of the evaluated dogs, primarily from bone marrow and spleen, which are recognized as reliable sampling sites despite limitations related to bacterial/fungal contamination. PCR allowed parasite detection in culture-negative samples, in agreement with established diagnostic evidence [17,19]. The identification of parasites in the aqueous humor of a single dog supports ocular tropism and may explain the severe pathology observed in this animal; however, this finding should be interpreted as an isolated observation rather than as evidence of the frequency or consistency of ocular tissue tropism.

Hematological alterations included anemia, thrombocytopenia, lymphopenia, monocytopenia, and variable neutrophilia, reflecting immune dysregulation commonly described in CVL. These findings are comparable to patterns reported by Durán-Galea et al. (2024) [44], who explored inflammatory indices such as the neutrophil-to-lymphocyte ratio (NLR) and systemic immune-inflammation index (SII). Although lymphocyte and neutrophil variations were observed in the present study, these indices were not calculated, and no prognostic interpretations can be made.

Biochemical abnormalities were also prominent in our cohort. Hypoalbuminemia and hyperglobulinemia were consistently present, consistent with hepatic involvement and chronic inflammation [37], as well as increased urea levels, suggesting renal impairment. Variations in aminotransferase activity were observed and are in line with previous reports describing hepatocellular alterations in CVL [45]; however, these parameters were not evaluated as markers of disease severity.

Although statistically significant differences were identified between dogs with and without ocular involvement for WBC count, neutrophil count, and AST levels, suggesting a potential association between the ocular lesions identified and systemic inflammation, these findings should be interpreted cautiously. Individual analysis revealed that a small number of dogs with ocular alterations presented markedly elevated values, disproportionately influencing group means. Dogs without ocular involvement exhibited more homogeneous reductions in leukocyte and neutrophil counts. Consequently, the data does not support a definitive association between systemic inflammation and ocular lesion development. At most, the findings suggest that systemic inflammatory responses may contribute to ocular alterations in some animals, but the sample size and study design preclude causal or mechanistic conclusions.

Overall, ocular alterations represent a relevant clinical finding within the spectrum of CVL observed in this cohort. Their coexistence with systemic changes underscores the multisystemic character of the disease; however, the present results do not support their use for clinical staging, prognostic assessment, or therapeutic decision-making. Further studies involving larger cohorts and longitudinal designs are necessary to clarify the clinical significance of ocular involvement in canine visceral leishmaniasis.

Future research incorporating serial ophthalmic assessments, quantitative inflammatory indices, and longitudinal biochemical monitoring may further elucidate the prognostic value of ocular lesions and deepen understanding of the immunopathogenic mechanisms that underlie both ocular and systemic CVL.

## 5. Conclusions

This study shows that CVL caused by *Leishmania infantum* in an endemic region of Brazil is associated with a complex multisystemic profile, characterized by overlapping clinical, ophthalmological, hematological, biochemical, parasitological, and histopathological alterations. Lymphadenopathy, cachexia, anemia, thrombocytopenia, hyperglobulinemia, and hypoalbuminemia were among the most frequently observed systemic findings, reflecting immune dysregulation and multi-organ involvement within this cohort.

Ocular alterations were commonly identified and often occurred in conjunction with systemic abnormalities. The presence of conjunctivitis, blepharitis, uveitis, keratitis, and keratoconjunctivitis sicca, together with histopathological identification of amastigotes in ocular and periocular tissues, underscores that ocular involvement constitutes a relevant component of the clinical spectrum observed in dogs with CVL. However, these findings should be interpreted as descriptive associations, as this study does not assess diagnostic performance, disease staging, or prognostic value.

Parasitological isolation and molecular confirmation of *L. infantum* highlight the usefulness of combining parasitological, molecular, and histopathological approaches for confirming infection, particularly in settings where culture sensitivity may be limited by contamination. Nonetheless, the present data do not allow conclusions regarding the superiority or predictive value of any single diagnostic method.

Hematological and biochemical alterations contributed to the overall characterization of the affected animals, reflecting patterns commonly reported in CVL. However, associations with disease severity, progression, or outcome cannot be established due to the cross-sectional design and limited sample size.

Overall, our findings reinforce the multisystemic nature of CVL and document the frequent coexistence of ocular and systemic alterations in naturally infected dogs from endemic areas. These results support the recognition of CVL not as a systemic disease but also as a relevant cause of ocular morbidity in dogs living in endemic regions. The incorporation of ophthalmological examination into routine veterinary practice may contribute to earlier clinical suspicion, improved clinical management, and strengthened disease surveillance strategies. Given the zoonotic relevance of CVL, early recognition of both ocular and systemic manifestations in dogs is essential for optimizing veterinary care and supporting public health interventions.

However, although ophthalmological findings represent an important component of the clinical spectrum of CVL, their role in early detection, clinical decision-making, or prognostic assessment cannot be established within the scope of the present study. Further investigations involving larger cohorts, longitudinal follow-up, and formal evaluation of diagnostic and prognostic performance are required to clarify the clinical significance of ocular involvement in canine visceral leishmaniasis. In addition, future studies should address the immunopathological mechanisms underlying ocular lesions and explore their potential value as prognostic markers of disease progression in both canine and human visceral leishmaniasis.

## Figures and Tables

**Table 1:** Means and Standard Deviations of Hematological and Biochemical analysis from Dogs Naturally Infected with *Leishmania infantum* in São Luís, Brazil.

	Quantitative Parameter	Mean ± σ, (*n* = 25)	Minimum	Maximum	Within Reference Values (*n/%*)	Below Reference Values (*n*/%)	Above Reference Values (*n*/%)
Erythrogram	RBC (×10^6^/µL)	3.89 ± 1.17	1.52	6.52	5.5–8.5 (2/8%)	23/92%	0
	Hemoglobin (g/dL)	7.79 ± 2.33	3.03	13.03	12–18 (1/4%)	24/96%	0
	HCT (%)	25.72 ± 7.716	10	43	37–55 (2/8%)	23/92%	0
	PLT (×10^3^/µL)	208,600 ± 116,429	60,000	513,000	200,000–900,000 (12/48%)	13/52%	0
Leukogram	WBC (/µL)	9170 ± 7284	1500	27,000	6000–17,000 (10/40%)	11/44%	4/16%
	Eosinophils (/µL)	285.4 ± 458.9	0	2232	100–1250 (14/56%)	10/40%	1/4%
	Band Neutrophils (/µL)	265.4 ± 772.0	0	3780	0–540 (22/88%)	0	3/12%
	Segmented Neutrophils (/µL)	6952 ± 5852	1170	23,142	3000–11,500 (13/52%)	7/28%	5/20%
	Lymphocytes (/µL)	1367 ± 1157	115	5130	1000–4800 (13/52%)	11/44%	1/4%
	Monocytes (/µL)	325.7 ± 470.8	0	1620	150–1350 (10/40%)	13/52%	2/8%
Biochemistry	AST (U/L)	43.04 ± 20.74	0	125	26–66 (22/88%)	2/8%	1/4%
	ALT (U/L)	35.85 ± 29.27	0	141	21–102 (18/72%)	6/24%	1/4%
	Creatinine (mg/dL)	0.61 ± 0.49	0.1	3	0.5–1.5 (13/52%)	9/36%	3/12%
	Urea (mg/dL)	38.57 ± 31.80	12.9	150.2	10–60 (21/84%)	0	4/16%
	Total proteins (g/dL)	7.08 ± 2.33	39	11.7	5.4–7.7 (9/36%)	6/24%	10/40%
	Albumin (g/dL)	1.98 ± 0.57	1	2.9	2.3–3.8 (10/40%)	15/60%	0
	Globulin (g/dL)	5.17 ± 1.83	2.7	9.1	2.3–5.2 (15/60%)	0	10/40%

RBC: Red Blood Cells, HCT: Hematocrit, PLT: Platelet, WBC: White Blood Cells, AST: Aspartate Transaminase, ALT: Alanine Transaminase.

**Table 2:** Comparison of hematological and biochemical parameters between dogs with and without ocular involvement naturally infected with *Leishmania infantum* in São Luís, Brazil.

	Quantitative Parameter	Mean ± σ, (*n* = 25)		*p*-Value
		Dogs with Ocular Involvement (*n* = 20)	Dogs Without Ocular Involvement (*n* = 5)	
Erythrogram	RBC (×10^6^/µL)	3.98 ± 0.93	3.54 ± 1.97	0.4646 ^a^
	Hemoglobin (g/dL)	7.96 ± 1.86	7.09 ± 3.94	0.4649 ^a^
	HCT (%)	26.30 ± 6.14	23.40 ± 13.01	0.4640 ^a^
	PLT (×10^3^/µL)	196,300 ± 106,773	257,800 ± 152,971	0.3005 ^a^
Leukogram	WBC (/µL)	10,383 ± 7570	4320 ± 3065	0.0489 ^b^*
	Eosinophils (/µL)	202.5 ± 222.6	617.0 ± 925.4	0.4955 ^b^
	Band Neutrophils (/µL)	325.7 ± 856.4	24.20 ± 27.17	0.6399 ^b^
	Segmented	7986 ± 6095	2817 ± 1509	0.0192 ^b^*
	Neutrophils (/µL)			
	Lymphocytes (/µL)	1512 ± 1218	788.2 ± 673.9	0.1483 ^b^
	Monocytes (/µL)	388.8 ± 507.8	73.40 ± 74.56	0.1134 ^b^
Biochemistry	AST (U/L)	47.08 ± 20.23	26.84 ± 15.05	0.0485 ^a^*
	ALT (U/L)	30.61 ± 20.33	56.82 ± 49.90	0.0723 ^a^
	Creatinine (mg/dL)	0.64 ± 0.54	0.52 ± 0.27	0.8536 ^b^
	Urea (mg/dL)	41.91 ± 34.82	25.22 ± 5.49	0.4535 ^b^
	Total proteins (g/dL)	6.77 ± 2.33	8.36 ± 2.08	0.2097 ^b^
	Albumin (g/dL)	1.88 ± 0.56	2.38 ± 0.49	0.0825 ^a^
	Globulin (g/dL)	4.97 ± 1.81	5.98 ± 1.90	0.2641 ^b^

^a^ Student’s *t*-test; ^b^ Mann–Whitney test; * *p* ≤ 0.05 RBC: Red Blood Cells, HCT: Hematocrit, PLT: Platelet, WBC: White Blood Cells, AST: Aspartate Transaminase, ALT: Alanine Transaminase.

## Data Availability

The original contributions presented in this study are included in the article/Supplementary Materials. Further inquiries can be directed to the corresponding author.

## Supplementary Materials

The following supporting information can be downloaded at: https://www.mdpi.com/article/10.3390/pathogens15020217/s1. Table S1: Clinical signs of Dogs Naturally Infected with *Leishmania infantum* in São Luís, Brazil; Table S2: Hematological analysis of Dogs Naturally Infected with *Leishmania infantum* in São Luís, Brazil; Table S3: Biochemical analysis of Dogs Naturally Infected with *Leishmania infantum* in São Luís, Brazil; Table S4: Distribution of diagnostic results per diagnostic method of dogs naturally infected with *Leishmania infantum* in São Luís, Brazil.
