# Supplementary material for: Ocular Involvement as a Key Marker of Systemic Disease in Dogs Naturally Infected with Leishmania infantum: Clinical, Laboratory, and Histopathological Insights
Source: Pathogens. 2026 Feb 14;15(2):217. doi: 10.3390/pathogens15020217 (PMC12943500; doi:10.3390/pathogens15020217)
Supplement: Supplementary file 1 [file pathogens-15-00217-s001.zip › pathogens-4074733-supplementary.pdf]

Supplementary Table S1- Clinical signs of Dogs Naturally Infected with *Leishmania infantum* in São Luís, Brazil.

	Animal																								
**Clinical signs**	01	02	03	04	05	06	07	08	09	10	11	12	13	14	15	16	17	18	19	20	21	22	23	24	25
Alopecia	X	X	X	X	X	-	X	X	X	X	X	X	-	X	X	X	-	X	X	X	X	X	X	-	-
Cachexia	X	X	X	X	X	X	X	X	X	X	X	X	X	X	-	X	X	X	X	X	X	X	X	X	X
Onychogryphosis/ onychodystrophy	X	X	X	X	-	-	X	X	X	X	X	-	-	X	-	-	-	X	-	X	X	X	X	-	-
Skin lesion	X	X	X	X	X	-	X	X	-	-	X	X	-	X	X	-	X	-	X	-	X	X	X	X	X
Lymphadenopathy	X	X	X	X	X	X	X	X	X	X	X	X	X	X	X	X	X	X	X	X	X	X	X	X	X
Hepatosplenomegaly	-	X	X	X	-	X	X	X	-	-	X	-	-	-	-	-	-	-	X	-	X	-	-	X	X
Ocular discharge (mucoid, purulent, or hemorrhagic)	X	X	X	-	-	X	X	X	X	X	-	X	X	X	X	X	-	-	X	-	X	X	X	X	X
Conjunctivitis	X	X	X	-	-	-	X	X	X	X	-	X	X	X	X	X	X	-	-	-	-	X	X	X	X
Blepharitis	-	-	X	-	-	-	-	-	-	X	-	X	-	-	-	X	X	-	X	-	-	-	X	-	-
Uveitis	-	X	-	-	-	-	X	X	-	X	-	-	-	-	-	-	-	-	-	-	-	-	-	-	-
Corneal opacity	-	-	-	-	-	-	-	X	-	-	-	X	-	-	-	-	-	-	-	-	-	-	-	-	-

Supplementary Table S2- Hematological analysis of Dogs Naturally Infected with *Leishmania infantum* in São Luís, Brazil.

	LEUKOGRAM						ERYTHROGRAM			
**Animal**	**Leukocytes (6,000-17,000)**	**Eosinophils (100-1,250)**	**Band Neutrophils (0-540)**	**Segmented Neutrophils (3,000-11,500)**	**Lymphocytes (1,000-4,800)**	**Monocytes (150-1,350)**	**Erythrocytes (5.5-8.5)**	**Hemoglobin (12-18)**	**Hematocrit (37-55)**	**Platelets (200,000-900,000)**
**1**	26,000 ↑	0 ↓	1,064 ↑	23,142 ↑	2,394	0 ↓	3.03 ↓	6.06 ↓	20 ↓	105,000 ↓
**2**	10,250	512.5	0	6,970	1,743	1,025	4.55 ↓	9.09 ↓	30 ↓	203,000
**3**	9,100	273	0	8,099	546 ↓	182	2.27 ↓	4.55 ↓	15 ↓	60,000 ↓
**4**	5,000 ↓	250	0	3,600	1,050	100 ↓	3.03 ↓	6.06 ↓	20 ↓	106,000 ↓
**5**	3,300 ↓	528	66	2,244 ↓	396 ↓	66 ↓	6.52	13.03	43	513,000
**6**	5,000 ↓	150	150	4,250	400 ↓	100 ↓	3.94 ↓	7.88 ↓	26 ↓	70,000 ↓
**7**	19,000 ↑	950	190	14,250 ↑	2,470	1,140	5.91	11.82 ↓	39	307,000
**8**	5,250 ↓	315	0	4,253	578 ↓	105 ↓	3.48 ↓	6.97 ↓	23↓	176,000 ↓
**9**	2,300 ↓	161	23	1,794 ↓	115 ↓	207	2.73 ↓	5.45 ↓	18 ↓	285,000
**10**	13,650	0 ↓	683 ↑	11,193	1,092	683	4.85 ↓	9.70 ↓	32↓	493,000
**11**	1,500 ↓	0 ↓	30	1,170 ↓	285 ↓	15 ↓	2.27 ↓	4.55 ↓	15 ↓	230,000
**12**	3,150 ↓	220.5	0	2,300 ↓	567 ↓	63 ↓	4.70 ↓	9.39 ↓	31 ↓	282,000
**13**	3,300 ↓	66 ↓	0	2,970 ↓	264 ↓	0 ↓	3.33 ↓	6.67 ↓	22 ↓	160,000 ↓
**14**	20,800 ↑	0 ↓	0	18,096 ↑	1,248	1,456 ↑	5.00 ↓	10.00 ↓	33 ↓	100,000 ↓
**15**	6,500	260	0	4,550	1,495	195	3.03 ↓	6.06 ↓	20 ↓	260,000
**16**	7,000	210	0	3,640	2,870	280	4.55 ↓	9.09 ↓	30 ↓	215,000
**17**	7,350	220.5	147	6,615	368 ↓	0 ↓	3.79 ↓	7.58 ↓	25 ↓	230,000
**18**	2,500 ↓	75 ↓	25	2,050 ↓	350 ↓	0 ↓	1.52 ↓	3.03 ↓	10 ↓	190,000 ↓
**19**	4,000 ↓	80 ↓	0	2,400 ↓	1,320	200	2.73 ↓	5.45 ↓	18 ↓	190,000 ↓
**20**	9,300	2,232 ↑	0	5,022	1,860	186	4.39 ↓	8.79 ↓	29 ↓	250,000
**21**	16,500	0 ↓	0	14,025 ↑	2,475	0 ↓	4.24 ↓	8.48 ↓	28 ↓	310,000
**22**	27,000 ↑	270	3,780 ↑	16,200 ↑	5,130 ↑	1,620 ↑	4.24 ↓	8.48 ↓	28 ↓	70,000 ↓
**23**	9,000	90 ↓	180	6,210	2,250	270	4.09 ↓	8.18 ↓	27 ↓	160,000 ↓
**24**	5,700 ↓	0 ↓	228	3,192	2,166	114 ↓	5 ↓	10 ↓	33 ↓	150,000 ↓
**25**	6,800	272	68	5,576	748 ↓	136 ↓	4.24 ↓	8.48 ↓	28 ↓	100,000 ↓

Note: ↓ Below reference values ↑ Above reference values

Supplementary Table S3- Biochemical analysis of Dogs Naturally Infected with *Leishmania infantum* in São Luís, Brazil.

Animal	AST (RV: 26 a 66 Ul/L)	ALT (RV: 21 a 102 Ul/L)	Creatinine (RV: 0.5 a 1.5 mg/dL)	Total Proteins (RV: 5.4 a 7.7 g/dL)	Albumin (RV: 2.3 a 3.8 g/dL)	Globulin (RV: 2.3 a 5.2 g/dL)	Urea (RV: 10 a 60 g/dL)
1	24.10 ↓	39.3	0.5	6	1.9 ↓	4.1	27.71
2	30.2	23.2	0.5	3.9 ↓	1.0 ↓	2.9	63.9 ↑
3	37.7	22.1	0.5	4.5 ↓	1.2 ↓	3.3	65.8 ↑
4	31.9	21	0.5	6.4	1.8 ↓	4.6	30.4
5	34.3	38.1	0.8	7	2.7	4.3	21.5
6	44.3	81.1	0.5	5.7	2 ↓	3.7	27.6
7	39.3	29.5	0.6	5.5	2 ↓	3.5	30.5
8	125 ↑	62	1.10 ↑	10.20 ↑	2.5	7.80 ↑	18.5
9	38	56	1	10.40 ↑	2.4	8 ↑	59.1
10	36	40	0.8	9.80 ↑	2.4	7.40 ↑	37.2
11	35	62	0.8	8.90 ↑	2.9	6 ↑	23.4
12	53	54	0.5	7.2	2.20 ↓	5	47.1
13	46	28	2.60 ↑	6.6	1.90 ↓	4.7	150.20 ↑
14	42	30	0.20 ↓	5.7	1.20 ↓	4.5	31.3
15	43	31	0.40 ↓	7.3	2.4	5	17.4
16	43	32	0.10 ↓	3.90 ↓	1.50 ↓	3.9	15.8
17	41	13↓	0.20 ↓	3.90 ↓	1.10 ↓	2.7	12.9
18	0 ↓	141 ↑	0.20 ↓	11.70 ↑	2.6	9.10 ↑	31.6

19	44	27	0.40 ↓	4.70 ↓	1.40 ↓	3.3	23.4
20	33	22	0.30 ↓	7.80 ↑	1.90 ↓	5.90 ↑	19.2
21	56	7↓	0.5	8.60 ↑	2.3	6.30 ↑	24.5
22	39.08	13↓	0.30 ↓	3.90 ↓	1.10 ↓	2.8	112.5 ↑
23	46	13↓	0.30 ↓	9.20 ↑	1.80 ↓	7.4 ↑	20.4
24	52	11↓	0.7	8.50 ↑	2.7	5.8 ↑	26.4
25	62	0 ↓	1.10 ↑	9.90 ↑	2.6	7.30 ↑	25.9

Note: ↓ Below reference values; ↑ Above reference values, AST: Aspartate Transaminase, ALT: Alanine Transaminase, RV: Reference value.

Supplementary Table S4 – Distribuition of diagnostic results per diagnostic method of dogs naturally infected with *Leishmania infantum* in São Luís, Brazil

Animal	Elisa/DPP	Parasite isolation	PCR	Schirmer's Test	Fluorescein	Bone marrow smear	Ocular histopathology
**1**	+/+	+	**	+	+	**	+
**2**	+/+	+	**	-	-	**	-
**3**	+/+	+	**	-	-	**	-
**4**	+/+	+	**	+	+	**	+
**5**	+/+	-	-	-	-	**	-
**6**	+/+	-	-	-	-	**	-
**7**	+/+	+	**	-	-	**	-
**8**	+/+	-	**	+	-	+	-
**9**	+/+	-	+	+	-	**	-
**10**	+/+	-	+	+	-	**	+
**11**	+/+	-	+	+	+	**	-
**12**	+/+	-	+	-	-	**	-
**13**	+/-	-	-	+	-	+	-
**14**	+/+	- / *	**	-	-	**	-

**15**	+/+	+	**	-	-	**	-
**16**	+/+	- / *	**	-	-	**	-
**17**	+/+	+	**	+	-	**	+
**18**	+/+	+	**	-	+	**	-
**19**	+/+	+	**	-	-	**	-
**20**	+/+	+	**	-	-	**	-
**21**	+/+	+	-	-	+	**	-
**22**	+/+	- / *	**	-	+	**	-
**23**	+/+	+	**	-	-	**	-
**24**	+/+	+	**	-	+	**	-
**25**	+/+	+	**	-	-	+	-

+ positive result; - negative result; *visualization of promastigote forms; ** not done
